# Effects of low-dose rapamycin on lymphoid organs of mice prone and resistant to accelerated senescence

**DOI:** 10.3389/fimmu.2024.1310505

**Published:** 2024-03-07

**Authors:** Rafael dos Santos Barros, Luiz Adriano Damasceno Queiroz, Josiane Betim de Assis, Kamilla Costa Pantoja, Sofia Xavier Bustia, Emanuella Sarmento Alho de Sousa, Stephen Fernandes Rodrigues, Eliana Hiromi Akamine, Anderson Sá-Nunes, Joilson O. Martins

**Affiliations:** ^1^ Laboratory of Immunoendocrinology, School of Pharmaceutical Sciences, Department of Clinical and Toxicological Analyses, University of São Paulo, São Paulo, Brazil; ^2^ Laboratory of Experimental Immunology, Institute of Biomedical Sciences, University of São Paulo, São Paulo, Brazil; ^3^ Laboratory of Vascular Nanopharmacology, Institute of Biomedical Sciences, University of São Paulo, São Paulo, Brazil; ^4^ Laboratory of Vascular Biology, Institute of Biomedical Sciences, University of São Paulo, São Paulo, Brazil

**Keywords:** aging, immunosenescence, rapamycin, immune response, T lymphocytes

## Abstract

Aging is a complex, natural, and irreversible phenomenon that subjects the body to numerous changes in the physiological process, characterized by a gradual decline in the organism’s homeostatic mechanisms, closely related to immunosenescence. Here, we evaluated the regulation of immunosenescence in lymphoid organs of senescence-accelerated prone 8 (SAM-P8) and senescence-accelerated resistant 1 (SAM-R1) mice treated with a low dose of rapamycin (RAPA). Mice were treated with a dose of 7.1 µg/kg RAPA for 2 months and had body mass and hematological parameters analyzed prior and during treatment. Cellular and humoral parameters of serum, bone marrow, thymus, and spleen samples were evaluated by ELISA, histology, and flow cytometry. Changes in body mass, hematological parameters, cell number, and in the secretion of IL-1β, IL-6, TNF-α, IL-7, and IL-15 cytokines were different between the 2 models used. In histological analyses, we observed that SAM-P8 mice showed faster thymic involution than SAM-R1 mice. Regarding the T lymphocyte subpopulations in the spleen, CD4^+^ and CD8^+^ T cell numbers were higher and lower, respectively, in SAM-P8 mice treated with RAPA, with the opposite observed in SAM-R1. Additionally, we found that the low dose of RAPA used did not trigger changes that could compromise the immune response of these mice and the administered dose may have contributed to changes in important lymphocyte populations in the adaptive immune response and the secretion of cytokines that directly collaborate with the maturation and proliferation of these cells.

## 1 Introduction

Aging is a natural, progressive, and irreversible process that is part of the life cycle. It is a complex phenomenon that affects the body in numerous aspects, including biological, physiological, environmental, psychological, behavioral, and social changes (1, 2). There are 2 main phases in aging: senescence and senility (3, 4). Senescence is characterized by healthy changes that accumulate over time without any recognized disease mechanism. Senility, on the other hand, is characterized by the onset of chronic diseases and disabilities that impair physiological functions and compromise life quality (4, 5).

Cellular senescence can be defined as a state in the cell cycle associated with various changes, such as the limitation of proliferation of compromised cells (6, 7). Although there is no single parameter for cellular senescence, several reliable markers are recognized (2, 8). One of them refers to the senescence-associated secretory phenotype (SASP), in which there is an excessive secretion of pro-inflammatory cytokines, such as IL-1β, IL-6, and TNF-α (9–12). This inflammatory process over time contributes to a chronic inflammatory state known as “inflammaging,” which is a pro-inflammatory phenotype that accompanies senility in mammals (2, 4, 6, 12).

The cells of the immune system are susceptible to the effects of senescence, known as immunosenescence (13). In this process, typical changes of senescence occur, together with dysfunction in the immune response and restructuring of the lymphoid organs (8, 14, 15). It is known that aging results in increased susceptibility to infections and diseases, decreased immune functionality, and altered distribution of immune cells, especially T lymphocytes, such as CD4^+^ T cell lymphopenia and weak immune response, as well as accumulation of CD8^+^ T cells (16–19). On the other hand, B lymphocytes are differently affected by age, presenting changes in the B cell compartment and scarcity of humoral immunity. Although the total number of mature B cells decreases with age, there are contradictory studies on the proportion of these cells in the naive and memory immune compartments (15, 17, 19, 20).

Several studies have correlated the delay of aging and cellular senescence with the effects of the drug rapamycin (RAPA) (21–23). RAPA is an immunosuppressant medication commonly used to prevent organ rejection in transplants, which also has antiproliferative effects, acting on the inhibition of the mammalian target of rapamycin (mTOR) protein and promoting cellular autophagy in a positive manner (21, 22, 24, 25). The mTOR protein is a serine-threonine kinase belonging to the TOR protein family, highly conserved in eukaryotic cells, and one of the main proteins responsible for protein synthesis and degradation, involved in nutrient sensing, metabolic regulation, and regulatory processes of autophagy (21, 26, 27).

The activity of RAPA begins with binding to the protein FKBP12, forming the RAPA-FKBP12 complex (25). This complex acts as an allosteric inhibitor of mTOR, resulting in the inhibition of the kinase activity of the mTOR protein, thus mimicking the scarcity of nutrients in the intracellular environment. This state of nutrient deprivation induces the initiation of the autophagic machinery, which may be involved in the anti-aging effect observed in the action of this drug (28, 29). Chemical mTOR inhibitors are currently the only pharmacological intervention known to increase lifespan in all experimental models tested, offering the prospect of therapeutically improving quality of life – and perhaps even lifespan – in clinical medicine (21, 22, 24, 25). To achieve this, however, it is necessary to define the exact mechanisms and tissues in which mTOR inhibition promotes longevity and reduces age-related diseases, mainly in the immune system, responsible for defending the body against external agents and internal defects, which tend to be more critical for the health and survival of the organism, when it starts to lose effectiveness at advanced ages (30).

In this sense, the use of RAPA as a viable alternative for anti-aging therapies must be carefully tested to evaluate its impact on the body’s immune system, given its immunosuppressive properties, which can be mitigated in interval doses as demonstrated by Arriola et al. (2015) – even though they still show an immunosuppressive effect when compared to control animals (31). This happens because the doses normally used to activate autophagy in aging studies are found to be in the range of 2 to 10 mg/kg, which may be high enough to maintain its antiproliferative effect in cells. However, studies such as that by Saegusa et al. (2020) show that even small doses such as 0.025 mg/kg of RAPA are capable of activating autophagy in mice in almost the same proportion as a dose 100 times higher than 2.5 mg/kg (32). Therefore, in this study, we aimed to evaluate the potential effects of a low dose of RAPA on the immunosenescent phenotype and the changes associated with this process in senescence-accelerated prone 8 (SAM-P8) and senescence-accelerated resistant 1 (SAM-R1) mice. For this end, we assessed how a low dose of RAPA impacts the lymphoid organs of these animals.

## 2 Materials and methods

### 2.1 Animals

Twelve-week-old SAM-P8 and SAM-R1 mice were used. The animals were maintained at 23 ± 2 °C, in mini-isolators, in a 12-hour light/dark cycle, with water and food available, undergoing an acclimation period of 7 days before starting RAPA treatment. Artificial devices were placed in the cages to enrich the environment (cotton, igloo, cardboard tubes, etc.). This study was evaluated and approved by the Ethics Committee on Animal Use (CEUA) at the School of Pharmaceutical Sciences (protocol number: CEUA/FCF/632) and at the Institute of Biomedical Sciences (protocol number: CEUA/ICB/4116171120), at the University of São Paulo, Brazil. All experiments were conducted in strict accordance with the principles and guidelines of the National Council for the Control of Animal Experimentation (CONCEA).

### 2.2 Measurement of body weight

The variation in animal body mass was monitored by a digital scale, once every 10 days during the 2 months of the study. The first evaluation occurred when the animals were 12 weeks old (referred to as day 0) and the last evaluation occurred on the day before euthanasia and biological sample collection (referred to as day 60), when the animals were 20 weeks old.

### 2.3 Study design and RAPA administration protocol

RAPA administration protocol was adapted from methodologies used in previous studies (31, 33, 34). The first work verified that administering RAPA at 5-day intervals did not interfere with glucose metabolism in C57BL/6 mice, unlike daily administration, which caused hyperglycemia and hyperinsulinemia (31). Xu et al. (2020) found that 12-week-old SAM-P8 and SAM-R1 male mice treated with RAPA for 2 months showed a decrease in the level of the malondialdehyde compound and an increase in superoxide dismutase enzyme activity, both related to oxidative stress. In the same study, the authors also demonstrated increased autophagy activity in SAM-P8 mice (33). Zhang et al. (2012) compared high and low doses of RAPA in the T lymphocyte response to ocular inflammation and concluded that low doses positively regulated the immune response, contributing to the proliferation, amplification, and prolongation of T cell response (34).

The animals were divided into 4 groups, namely: SAM-R1 Control, SAM-R1 RAPA, SAM-P8 Control, SAM-P8 RAPA. In the treated groups, animals received a dose of 7.1 µg/kg of RAPA (InvivoGen, Toulouse, France) per mouse, diluted in 150 µL of filtered water. The control group animals received only vehicle solution (filtered water) instead of the drug. The administration was performed by gavage, once every 5 days, for 2 months.

### 2.4 Collection and processing of biological samples

Mice were euthanized with isoflurane and had their left femur and tibia removed. The bone marrow was washed with 1 mL of RIPA buffer (50 mM Tris, pH 8.0, 150 mM NaCl, 1% Triton X-100, 0.1% SDS supplemented with protease inhibitor tablets) and stored in Eppendorf tubes. The thymus and spleen were collected, stored in liquid nitrogen, and stored in 1.5 mL conical tubes. Subsequently, the samples were processed using a Polytron homogenizer (Polytron^®^ PT 2500, Thomas Scientific, New Jersey, USA). The supernatants were stored at 80°C until future analyses of protein quantification and cytokines.

### 2.5 Evaluation of hematological parameters

Blood collections were performed at 2 distinct moments, before starting the RAPA treatment and after the animals were euthanized, respectively by caudal and cardiac puncture. The whole blood collected was stored in tubes containing 10% EDTA and analyzed in the automated hematology counter (BC-2800Vet Mindray, Shenzhen, GD, China) to determine the following hematological parameters: red blood cells (RBC), hemoglobin (HGB), hematocrit (HCT), mean corpuscular volume (MCV), mean corpuscular hemoglobin (MCH), mean corpuscular hemoglobin concentration (MCHC), red cell distribution width (RDW), leukocytes (WBC), lymphocytes (Lymph), monocytes (Mon), granulocytes (Gran), and the percentage of lymphocytes (Lymph%), monocytes (Mon%), and granulocytes (Gran%). We compared the results generated with the reference values provided by the hematology analyzer (Mindray^®^ Animal Care) (**Supplementary Table 1**).

### 2.6 Cytokine measurements

The protein content of serum, bone marrow, thymus, and spleen homogenates was quantified using the BCA Protein Assay kit (Thermo Fisher Scientific, Massachusetts, USA), according to the manufacturer’s recommendations. The cytokines IL-1β, IL-6, and TNF-α were measured in all samples, while the cytokines IL-7 and IL-15 were analyzed only in bone marrow samples. Cytokine measurements were performed using DuoSet^®^ ELISA (R&D Systems, Minnesota, USA), according to the manufacturer’s instructions. Absorbance readings were taken on the Epoch microplate spectrophotometer (Agilent Technologies, California, USA). The cytokine concentration was adjusted to the protein content of each sample.

### 2.7 Histological analysis

Samples from the bone marrow, thymus, and spleen were collected and placed in tubes containing 10% formalin solution for fixation. After 24 hours of fixation, the materials were dehydrated in 70% ethanol, cleared in xylene, and embedded in paraffin blocks. After embedding, cross-sectional tissue sections of approximately 5 μm thickness were obtained and subsequently stained with hematoxylin and eosin (HE). The histological slides were analyzed by light microscopy. General changes in organ structures were evaluated qualitatively, according to the guidelines by Elmore S. A (2006) for histological evaluation of the thymus (35), spleen (36), and bone marrow (37).

### 2.8 Cellularity of the thymus and spleen

Following euthanasia, the entire thymus and spleen were aseptically collected in a laminar flow cabinet and maintained in RPMI 1640 medium (Thermo Fisher Scientific). The organs were mashed through 40 μm pore size cell strainers using a syringe plunger, centrifuged at 239 × *g*, 4 °C, for 5 minutes, and the supernatant was discarded. The red blood cells were lysed with ACK Lysing Buffer (Thermo Fisher Scientific) and the cells were stored in PBS containing 2% fetal bovine serum (FBS). Subsequently, the cells were diluted in Turk’s solution and cell counting was performed in a Neubauer chamber.

### 2.9 Flow cytometry

A suspension containing 2 × 10^6^ cells/mL was prepared in PBS and distributed into 96-well round-bottom plates. After centrifugation, the supernatant was discarded and the cells were stained with the viability reagent LIVE/DEAD™ (Thermo Fisher Scientific) and incubated for 10 minutes at 4 °C in the dark. After washes, the cells were incubated for 30 minutes, under the same conditions, with the fluorochrome-conjugated monoclonal antibodies anti-CD3-FITC (clone: 145-2c11), anti-CD19-APC (1D3), anti-CD4-APC-Cy7 (GK1.5), and anti-CD8-PE-Cy5 (53-6.7) (BD Biosciences). After rinsing, the cells were fixed and permeabilized using Foxp3/Transcription Factor Staining Buffer Set (eBioscience, Carlsbad, California, USA) and stained for 1 hour with anti-Foxp3-PE (FJK16s) monoclonal antibody according to the manufacturer’s instructions. The samples were washed and resuspended in PBS 2% FBS, transferred to polypropylene tubes (12 × 75 mm), and acquired by a flow cytometer (LSRFortessa™ X-20, BD Biosciences). Dot plot gating was performed based on cell size (forward scatter) and granularity (side scatter) for lymphocytes, dead cells were excluded (**Supplementary Figure 1**), and the results were analyzed using FlowJo software (version 7.5.5, BD Biosciences).

### 2.10 Lymphoproliferation

A suspension containing 1 × 10^7^ spleen cells/mL was prepared and distributed into 96-well flat-bottom plates. The cells were cultured in complete medium only (control) or stimulated with 1 µg/mL concanavalin A (Con A). The plates were maintained in a CO_2_ incubator at 37°C for 72 hours. After the first 48 hours of culture, 25 µL of 0.01% resazurin were added to the wells, and at the end of 72 hours, absorbance was analyzed as previously described (38, 39).

### 2.11 Myelogram

The femoral and tibial bones were removed, and the bone marrow cavity was washed with 3 mL of RPMI 1640 medium. The samples were then centrifuged at 300 × *g*, 4 °C, for 5 minutes, and the supernatant was discarded. The red blood cells were lysed with ACK Lysing Buffer (Thermo Fisher Scientific) and the content was resuspended in 1 mL of medium. Subsequently, the samples were diluted in Turk’s fluid, with cell counting performed in a Neubauer chamber, and a cell suspension containing 4 × 10^4^ cells/mL was prepared. Then, slides were prepared on a CytoSpin™ centrifuge (Thermo Fisher Scientific), with a rotation program of 400 × *g*, 4 °C, for 5 minutes. After drying the slides, they were stained with the rapid panoptic kit (Laborclin, Paraná, Brazil) and analyzed by light microscopy, with the counting of 100 cells per field, based on morphological criteria, classified as either mononuclear or polymorphonuclear (40, 41).

### 2.12 Statistical analysis

Data were evaluated by analyses of variance (ANOVA) and Tukey as a post-test on GraphPad Prism 8.0 software (GraphPad Software, San Diego, California, USA). Data were represented as mean ± standard error and values of *p <*0.05 (* or ^+^), *p <*0.01 (**), *p <*0.001 (***), and *p <*0.0001 (****) were considered significant.

## 3 Results

### 3.1 Measurement of body weight

**Figure 1:**
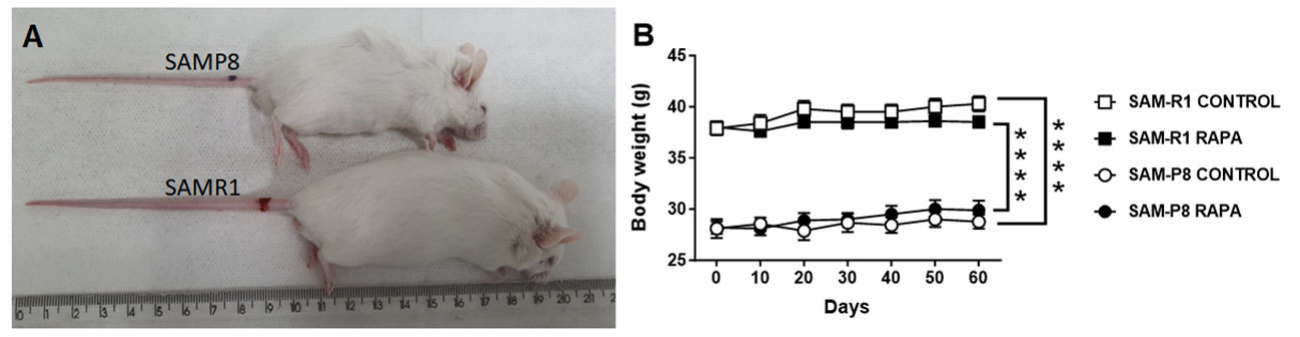
Body weight evolution in SAM-R1 and SAM-P8 mouse strains treated or not with rapamycin (RAPA). **(A)** image of adult SAM-R1 and SAM-P8 mice; **(B)** body weight. Animals were weighed at 10-day intervals, starting at 12 weeks old (day 0) until they reached 20 weeks old (day 60). RAPA treatment was performed as described in Material and Methods. *n* = 10 mice per group. ****p < 0.0001.

The body weight of the animals was monitored once every 10 days, with the first measurement taken when the animals were 12 weeks old (day 0) and the last measurement taken when the animals were 20 weeks old (day 60). It is important to highlight that SAM-R1 mice are bigger in size and weight than SAMP-P8 mice at 12 weeks old, with a difference of ~10 g between the strains (**Figures 1A, B**). Because the experiments were performed with fully developed adult animals, both the SAM-R1 and SAM-P8 mice displayed only a minor increase in weight (~1.5 g) between the beginning and end of the experiment (**Figure 1B**). In addition, the treatment with a low dose of RAPA did not statistically change the body weight of the animals in any group (**Figure 1B**).

### 3.2 Hematological parameters

**Figure 2:**
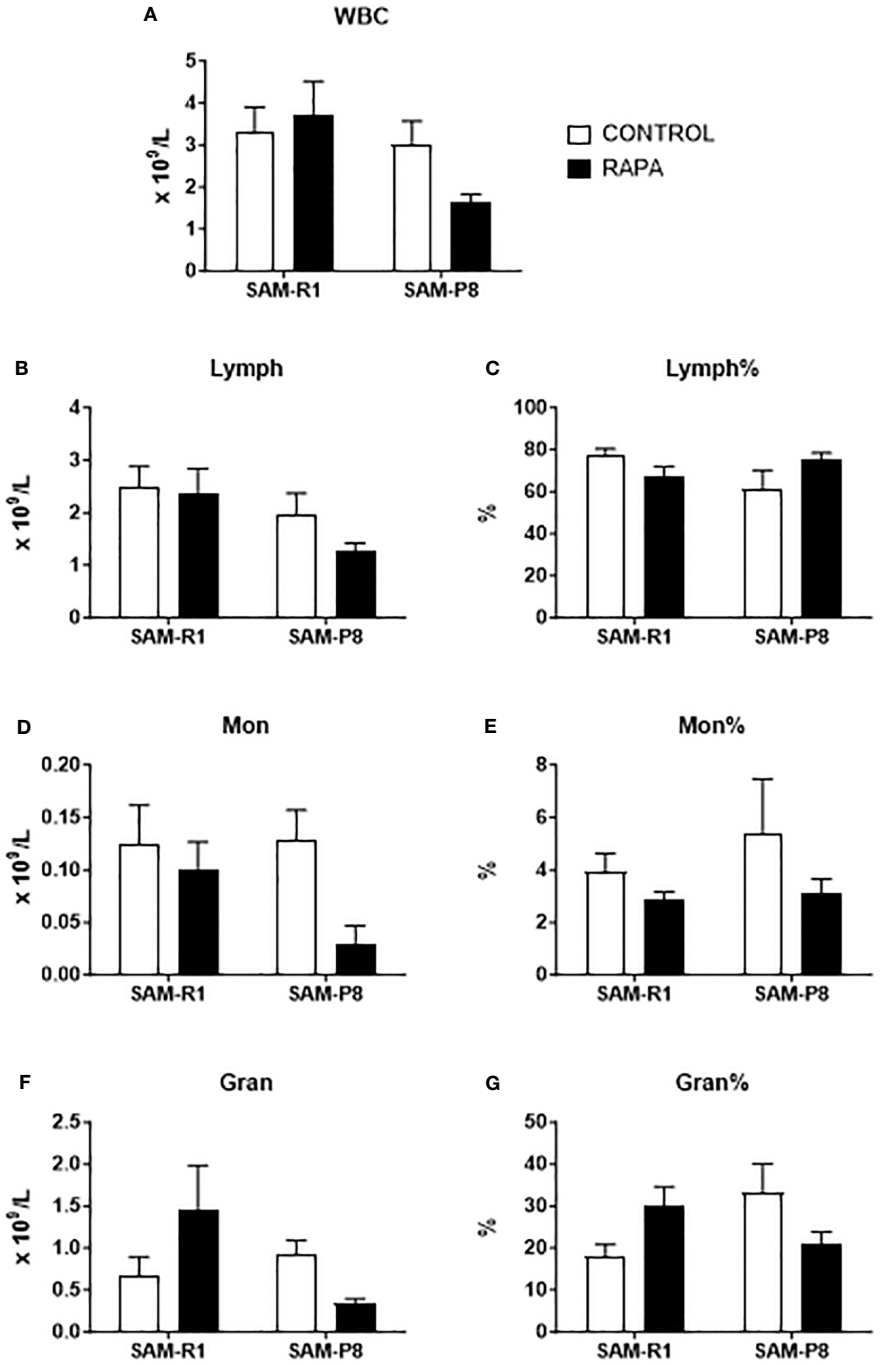
Leukogram of SAM-R1 and SAM-P8 mice. **(A)** total white blood cells (WBC); **(B)** total lymphocytes (Lymph); **(C)** percentage of lymphocytes (Lymph%); **(D)** total monocytes (Mon); **(E)** percentage of monocytes (Mon%); **(F)** total granulocytes (Gran); **(G)** percentage of granulocytes (Gran%). Hemogram analyses were performed after the euthanasia of the mice (animals at 20 weeks old). *n* = 8 (SAM-R1 CONTROL/RAPA groups); *n* = 7 (SAM-P8 CONTROL/RAPA groups).

**Figure 3:**
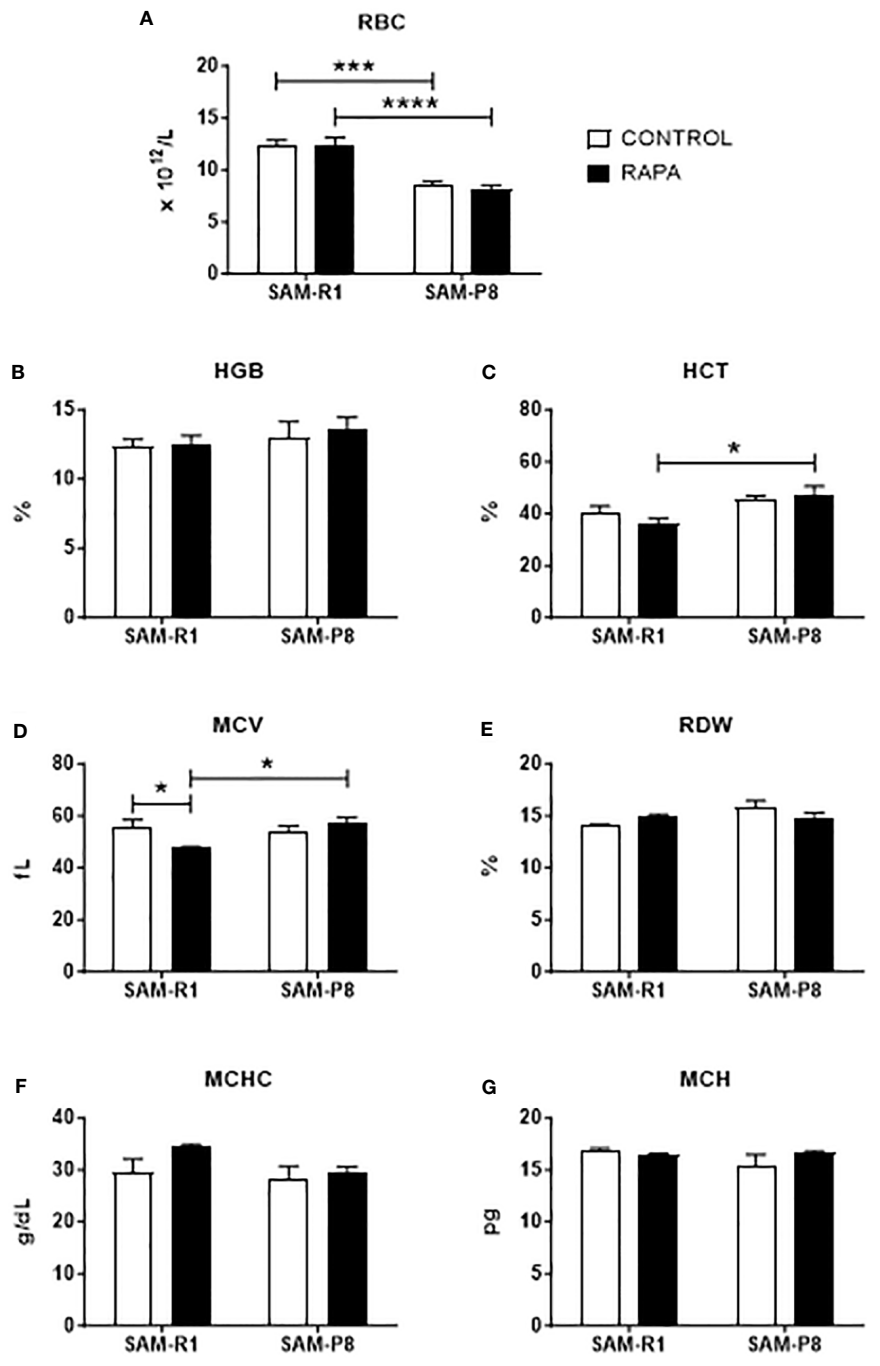
Erythrogram of SAM-R1 and SAM-P8 mice. **(A)** red blood cells (RBC); **(B)** percentage of hemoglobin (HGB); **(C)** hematocrit (HCT); **(D)** mean corpuscular volume (MCV); **(E)** red cell distribution width (RDW); **(F)** mean corpuscular hemoglobin concentration (MCHC); **(G)** mean corpuscular hemoglobin (MCH). Hemogram analyses were performed after the euthanasia of the mice (animals at 20 weeks old). *n* = 8 (SAM-R1 CONTROL/RAPA groups); *n* = 7 (SAM-P8 CONTROL/RAPA groups). *p < 0.05, ***p < 0.001 and ****p < 0.0001.

The leukocyte and erythrocyte series were evaluated at 2 different times: a day before the beginning of treatment (**Supplementary Figures 2**, **3**) and at the end of the treatment (**Figures 2**, **3**). At first, we observed that these strains show initial differences before treatment, with SAM-R1 having higher absolute values for WBC, Lymph, Mon, Gran, and MCHC, in addition to a higher percentage of RDW, than SAM-P8. Additionally, SAM-R1 presents a higher percentage of MCV values than SAM-P8 (**Supplementary Figures 2**, **3**). Such differences virtually disappeared upon RAPA treatment. The only few differences observed were: 1) a decrease in RBC absolute values in SAM-P8 when compared to SAM-R1 mice under both control and RAPA conditions (**Figure 3A**); 2) a reduction in MCV in SAM-R1 animals under RAPA treatment (**Figure 3D**). Furthermore, it is worth highlighting that the average population of RBC in SAM-R1 control in the beginning of the experiment (pre-treatment) was elevated compared to the reference value for mice (**Supplementary Table 1**). The 2 strains analyzed before treatment showed a concentration of HGB also above the expected reference values. Results of MCH in the SAM-P8 RAPA pre-treatment group and RDW in the SAM-R1 RAPA post-treatment group also presented above-reference values. In the leukogram, we noticed that the population of WBC in the SAM-R1 lineage before treatment showed results above the reference range.

### 3.3 Cytokine measurements

**Figure 4:**
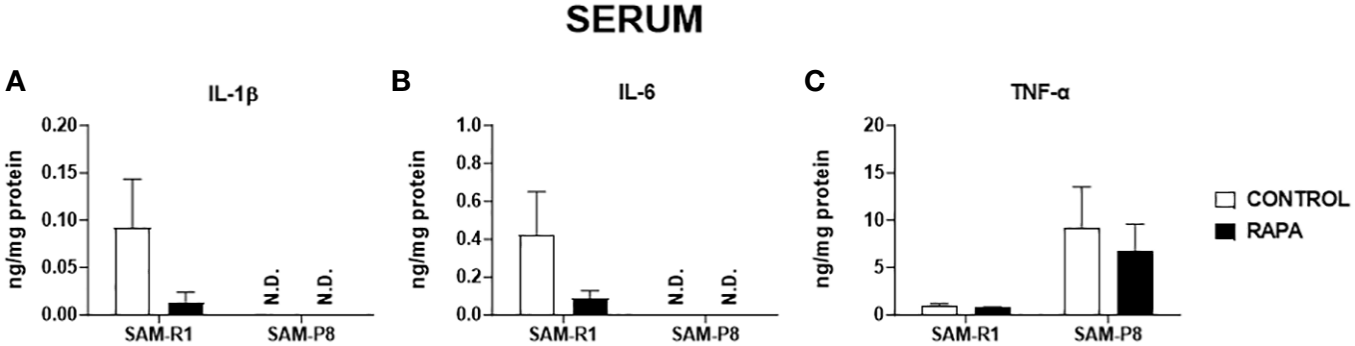
SASP-related cytokines in serum of SAM-R1 and SAM-P8 mice. **(A)** IL-1β; **(B)** IL-6; and **(C)** TNF-α. The measurements were performed in samples collected from 20-week-old control and RAPA-treated mice for 2 months. N.D: not detected. *n* = 5 mice per group.

**Figure 5:**
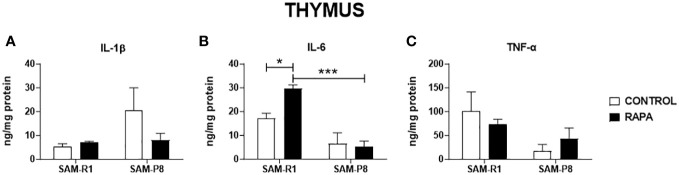
SASP-related cytokines in thymus of SAM-R1 and SAM-P8 mice. **(A)** IL-1β; **(B)** IL-6; and **(C)** TNF-α. The measurements were performed in samples collected from 20-week-old control and RAPA-treated mice for 2 months. *n* = 5 mice per group. *p < 0.05 and ***p < 0.001.

**Figure 6:**
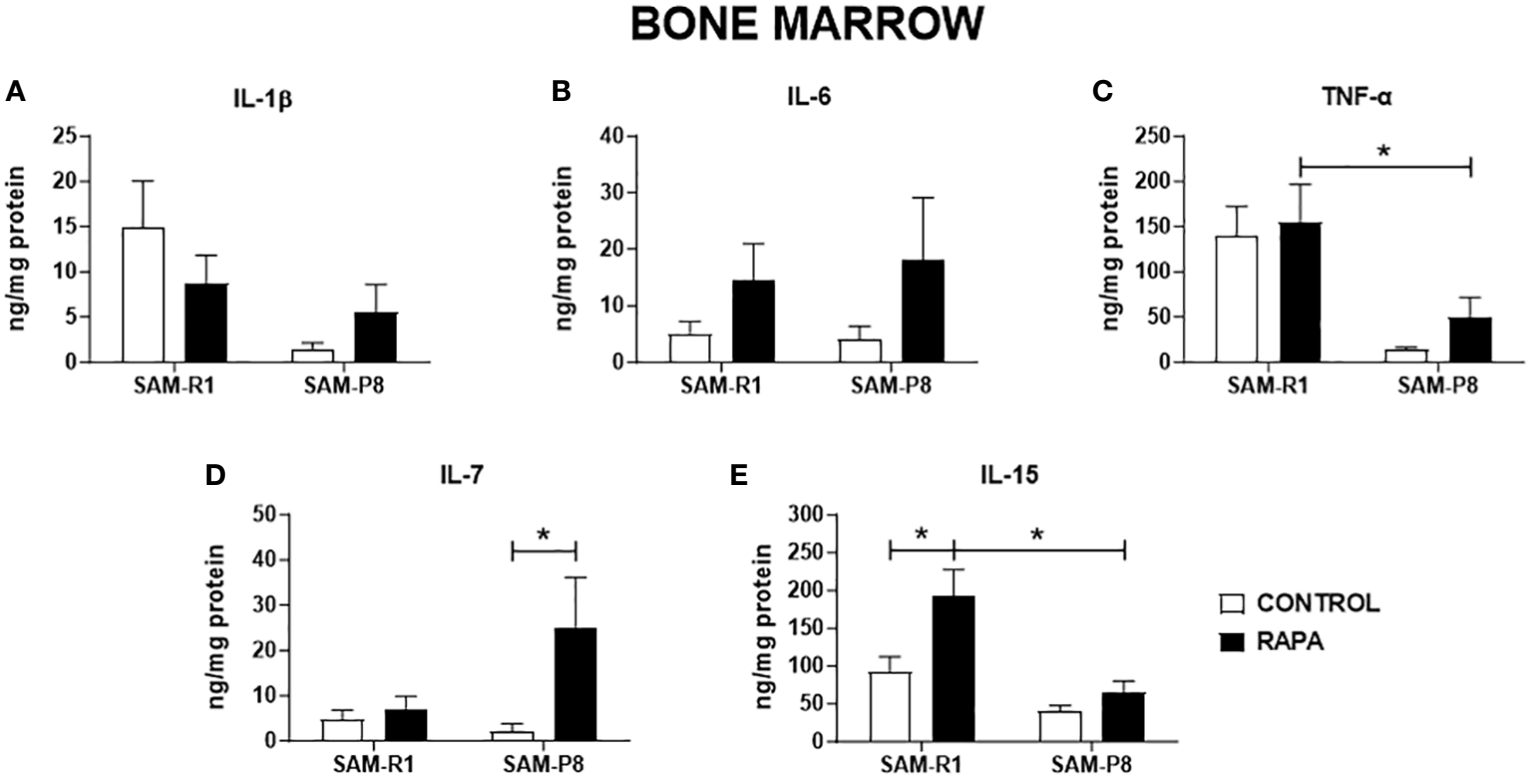
SASP-related cytokines in bone marrow of SAM-R1 and SAM-P8 mice. **(A)** IL-1β; **(B)** IL-6; **(C)** TNF-α; **(D)** IL-7; and **(E)** IL-15. The measurements were performed in samples collected from 20-week-old control and RAPA-treated mice for 2 months. *n* = 5 mice per group. *p < 0.05.

**Figure 7:**
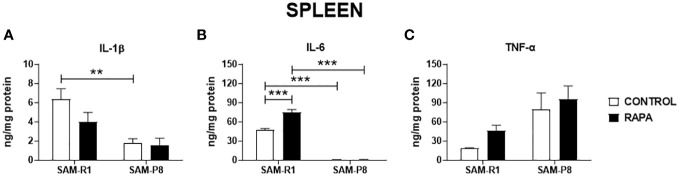
SASP-related cytokines in spleen of SAM-R1 and SAM-P8 mice. **(A)** IL-1β; **(B)** IL-6; and **(C)** TNF-α. The measurements were performed in samples collected from 20-week-old control and RAPA-treated mice for 2 months. *n* = 5 mice per group. **p < 0.01 and ***p < 0.001.

Next, we evaluated the SASP-related cytokines in serum and tissue homogenate samples. Regarding IL-1β, no significant differences were observed between the strains or following RAPA treatment in serum (**Figure 4A**), thymus (**Figure 5A**), or bone marrow (**Figure 6A**). However, SAM-R1 mice presented higher levels of IL-1β in the spleen when compared to SAM-P8 mice, although the RAPA treatment did not change either basal level of the cytokine (**Figure 7A**). For IL-6, treatment with RAPA significantly increased its levels in the thymus (**Figure 5B**) and spleen (**Figure 7B**) of SAM-R1 animals when compared to their control group, but not in serum (**Figure 4B**). In addition, the secretion of this cytokine was greater in SAM-R1 animals when compared to SAM-P8 in thymus, spleen and bone marrow, although RAPA treatment had no effect on IL-6 levels in the latter strain (**Figures 5B**, **6B**, **7B**). Regarding TNF-α, the bone marrow of the SAM-R1 control group showed significantly higher levels of the cytokine than the SAM-P8 control group (**Figure 6C**), with no differences in serum, thymus, or spleen (**Figures 4C**, **5C**, **7C**). RAPA treatment did not alter the basal levels of IL-6 in any strain or compartment. We noticed an increase of IL-7 in RAPA-treated SAM-P8 (**Figure 6D**) and IL-15 in SAM-R1 (**Figure 6E**) under RAPA treatment when compared to their respective control groups.

### 3.4 Histological analysis

**Figure 8:**
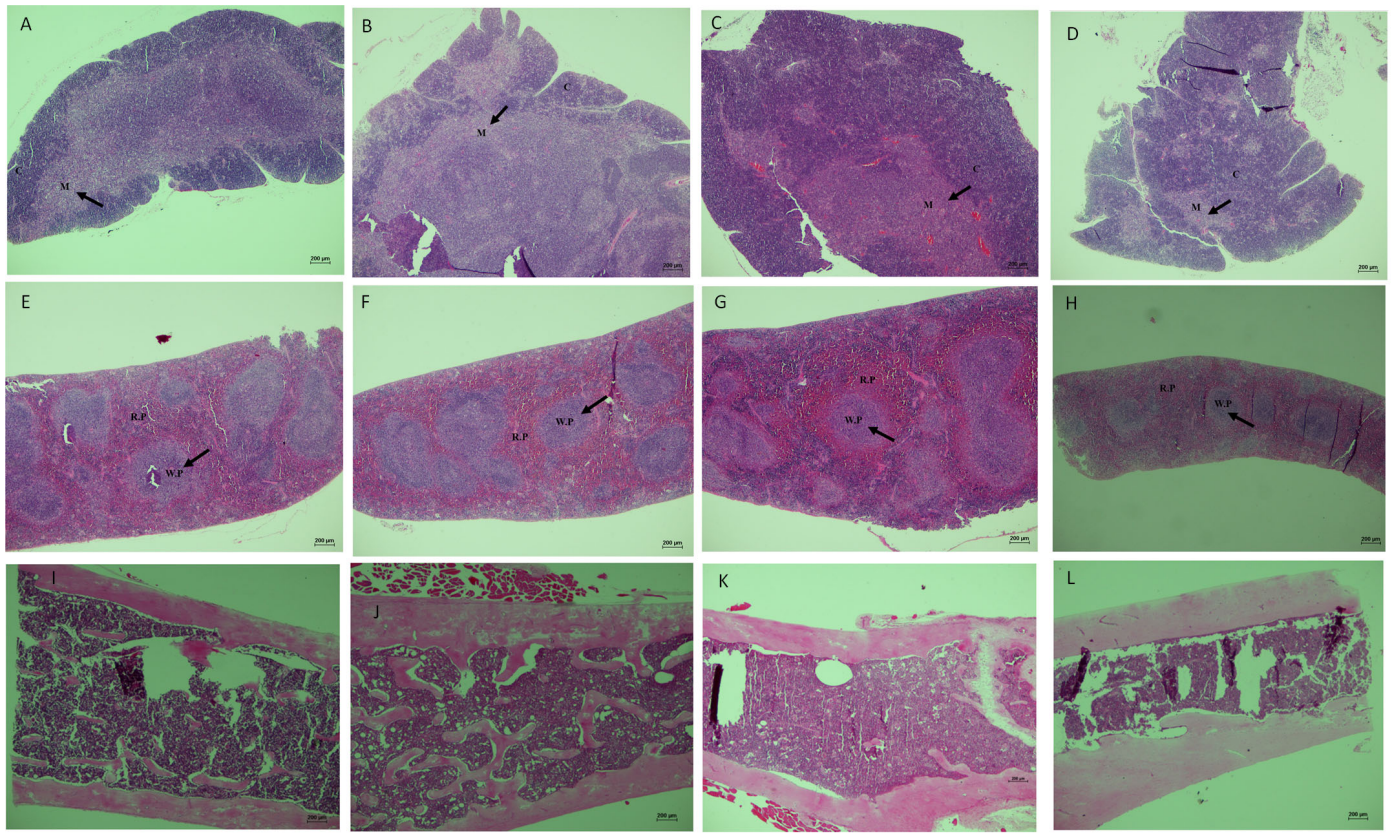
Histological evaluation of lymphoid organs of SAM-R1 and SAM-P8 mouse strains. **(A)** thymus of SAM-P8 mouse treated with RAPA; **(B)** thymus of SAM-P8 mouse control; **(C)** thymus of SAM-R1 mouse treated with RAPA; **(D)** thymus of SAM-R1 mouse control; **(E)** spleen of SAM-P8 mouse treated with RAPA; **(F)** spleen of SAM-P8 mouse control; **(G)** spleen of SAM-R1 mouse treated with RAPA; **(H)** spleen of SAM-R1 mouse control; **(I)** bone marrow of SAM-P8 mouse treated with RAPA; **(J)** bone marrow of SAM-P8 mouse control; **(K)** bone marrow of SAM-R1 mouse treated with RAPA; **(L)** bone marrow of SAM-R1 mouse control. Microscopic images captured from paraffin-embedded histological slides, in longitudinal sections, stained with HE and magnified at 40 ×. C: cortical zone of the thymus; M: medullary zone of the thymus; WP: white pulp of the spleen; RP: red pulp of the spleen. The analysis was performed in samples collected from 20-week-old control and RAPA-treated mice for 2 months. *n* = 5 mice per group.

We observed significant differences in the organization of the thymic structures between the mice strains. In SAM-P8 mice, the thymic medullary region was found in a smaller proportion compared to the cortical zone (**Figures 8C, D**). However, there were no differences between the control and the RAPA-treated groups. On the other hand, in SAM-R1 mice, the cortical region occupied a smaller area than the medullary region (**Figures 8A, B**), and we also observed no differences in thymic structures between the control and RAPA-treated groups. Regarding the splenic and medullary tissues, no significant structural difference was found between the groups or mouse strains (**Figures 8E–L**).

### 3.5 Cellularity of the thymus, spleen, and bone marrow

We found that the cellularity of the thymus and spleen showed significant differences between the 2 strains of mice (**Table 1**). When comparing only the SAM-P8 groups, we observed a higher number of bone marrow and splenic cells in the RAPA-treated group, but with no statistical difference. Conversely, thymic cells are more abundant in the control group compared to the RAPA-treaded group, but with no statistical difference. Between the 2 groups of SAM-R1 mice, there is a lower number of bone marrow and thymic cells in the control group and increased cellularity in the spleen of the RAPA group, but again with no statistical difference.

**Table 1:** Evaluation of lymphoid organ cellularity in SAM-R1 and SAM-P8 mouse strains.

	SAM-R1		SAM-P8	
Parameter	Control (*n *= 4)	RAPA (*n *= 6)	Control (*n *= 4)	RAPA (*n *= 6)
**Bone marrow** **(×10^7^/mL)**	1.87 ± 0.6	1.74 ± 0.13	1.94 ± 0.33	2.52 ± 1.09
**Spleen** **(×10^7^/mL)**	9.18 ± 0.59^(*)^	11.15 ± 1.18**^(+)^**	2.62 ± 0.37^(*)^	3.06 ± 0.32**^(+)^**
**Thymus** **(×10^7^/mL)**	6.02 ± 0.33^(*)^	4.50 ± 0.69**^(+)^**	1.18 ± 0.21^(*)^	1.06 ± 0.33**^(+)^**

Results are expressed as mean values ± SEM. Cellularity was determined in bone marrow, spleen, and thymus samples from 20-week-old mice, both control and treated with rapamycin for 2 months. n: number of animals per group. Statistical representation was performed using * or +, interpreted as follows: results with the same symbols have statistical difference (p < 0.05) between the analyzed group(s) in relation to the same evaluated parameter.

### 3.6 Lymphoproliferation

**Figure 9:**
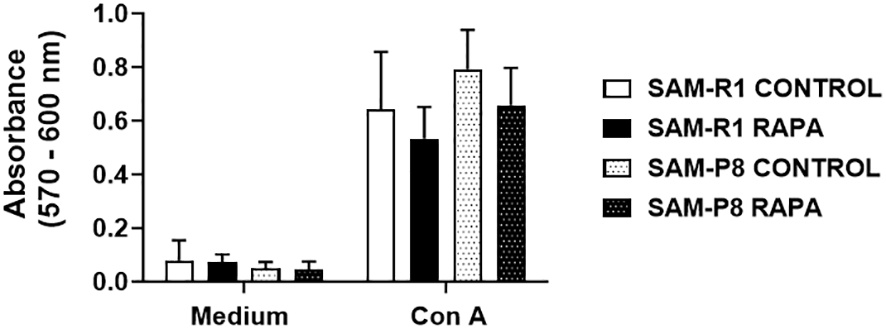
Splenic lymphocyte proliferation in SAM-R1 and SAM-P8 mice. Lymphocyte proliferation assay was stimulated by Con A (1 µg/mL) in spleen cells from 20-week-old control and RAPA-treated mice for 2 months. The lymphoproliferation result was determined by the absorbance value. *n* = 4 (control groups); *n* = 6 (RAPA groups).

We observed that ConA-stimulated cells from all groups proliferated in a similar manner. Although a slight increase in proliferation in the control groups compared to the RAPA-treated mice was observed, no statistical difference was reached (**Figure 9**).

### 3.7 Flow cytometry

**Figure 10:**
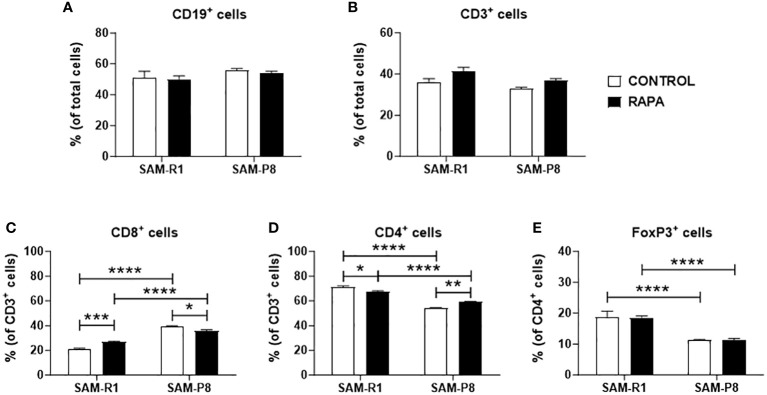
Spleen lymphocyte populations and subpopulations. **(A)** B lymphocyte population; **(B)** T lymphocyte population; **(C)** CD8^+^ T lymphocyte subpopulation; **(D)** CD4^+^ T lymphocyte subpopulation; **(E)** regulatory T lymphocyte subpopulation. Samples were obtained from 20-week-old control and RAPA-treated mice for 2 months. Total spleen cells were labeled with fluorochrome-conjugated antibodies and acquired by a flow cytometer. Analysis was performed using FlowJo (version 7.5.5) and is shown as a percentage (%) of cells. *n* = 4 (control groups); *n* = 6 (RAPA groups). *p < 0.05, **p < 0.01, ***p < 0.001, and ****p < 0.0001.

No significant differences were observed in the percentage of total B cell (**Figure 10A**) or T cell (**Figure 10B**) populations from spleen among the experimental groups. Nonetheless, when T cell subpopulations were analyzed, we observed that SAM-P8 animals present a higher basal percentage of splenic CD8^+^ T cells when compared to SAM-R1 (**Figure 10C**). Conversely, the proportion of CD4^+^ T cells in the spleen is higher in SAM-R1 mice than in SAM-P8 mice (**Figure 10D**). This difference in the CD4^+^ compartment seems to be related to the higher percentage of regulatory T cells (Treg or FoxP3^+^ cells) in SAM-R1 mice (**Figure 10E**). Interestingly, the chronic treatment with RAPA induced changes in the proportion of T cell subpopulations. RAPA-treated SAM-R1 mice had an increase in the CD8^+^ T cell population while SAM-P8 animals showed the opposite phenotype (**Figure 10C**). On the other hand, the CD4^+^ T cell population showed a small decrease in percentage in SAM-R1 mice upon RAPA treatment, but an increase in SAM-P8 mice upon treatment (**Figure 10D**). However, RAPA treatment induced no changes in splenic Treg population of either strain (**Figure 10E**).

### 3.8 Myelogram

**Figure 11:**
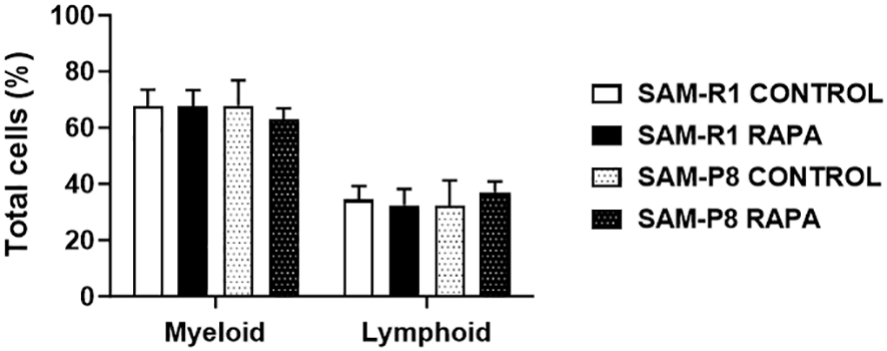
Myeloid and lymphoid lineages in SAM-R1 and SAM-P8 mice. Analysis of bone marrow smears from 20-week-old control and RAPA-treated mice for 2 months. The results are shown as the percentage (%) of cells, based on the counting of 100 cells per field. *n* = 5 mice per group.

Evaluation of the myelogram was performed with the aim of determining the quantity of progenitor cells in the mice studied. We found that the percentage of myeloid progenitor cells was higher than the lymphoid lineage in all groups evaluated, as shown in **Figure 11**.

## 4 Discussion

The SAM-P8 mouse strain is a well-established murine model for aging-related studies and presents disorders associated with senescence. This model, originally generated from AKR/J mice, was developed in 1981 (42). Due to its characteristics of accelerated senescence when compared to the SAM-R1 strain, the SAM-P8 is an excellent model for our study, as it is possible to verify the pronounced acceleration of the effects of the expression of the senescent phenotype in the aging process in mammals. Additionally, this strain displays a variety of pathological phenotypes that are associated with the senescent state, such as impairment of immune response, mitochondrial dysfunction, accumulation of oxidative damage, senile amyloidosis, deficits in learning and memory, alteration of the circadian cycle, and reduced lifespan (42, 43).

The range of RAPA administration doses varies widely depending on the route chosen and focus of study, ranging from 0.3 μg/kg to 50 mg/kg (44). In terms of blood concentration, levels above 15 ng/mL are considered high doses, while low doses are below this concentration (44). The most common side effects associated with high doses of RAPA are gastrointestinal, hematologic, and metabolic disturbances, as well as excessive immunosuppression (34, 44). However, on some occasions, the immunosuppressive effect can be positive and is used in clinical management aimed at avoiding tissue transplant rejection and in the treatment of autoimmune diseases (45–47). In addition, RAPA’s antiproliferative effects have been studied as a strategy against aging, neoplasms, and in neurodegenerative diseases such as Alzheimer’s and Parkinson’s (46, 48, 49).

In our study, we evaluated the evolution of mouse body mass over 2 months of RAPA treatment. No differences were observed between the respective control and treated groups, showing that the treatment protocol chosen did not affect weight maintenance, which has been previously described (50). During the aging process, the cellular composition of peripheral blood undergoes changes in the proportion of its populations (23). It has been demonstrated that young and elderly C57BL/6 mice treated with RAPA at 14 mg/kg were unable to display hematological changes caused by RAPA, only changes between the young and elderly phenotype (23). Similar results were found by our group, with the exception of MCV, which was reduced in the SAM-R1 RAPA-treated group, which could indicate a change in the size of the red blood cells of these animals in relation to their control. This putative effect of RAPA on erythropoiesis was already described in Wistar rats treated with 1 mg/kg of RAPA, 3 times a week for 4 months (51). Furthermore, comparing our findings with the results of Santos et al. (2016), who evaluated the blood count of C57BL/6, BALB/c, and Swiss mice at 12 weeks of age, we observed that SAM-P8 and SAM-R1 mice have higher values for the 3 hematimetric indices highlighted above. Thus, we suggest that our animal model studied also presents differences with other mice commonly used in scientific research (52).

Another problem resulting from senescence concerns is the secretion of inflammatory cytokines associated with SASP (10). These inflammatory cytokines contribute to a chronic state of systemic inflammation called inflammaging, which is one of the main risk factors for the development of age-related pathologies (2, 53). In our study, we also aimed to verify the baseline inflammatory activity state of these cytokines in serum and different tissues without the need to promote challenges to mice with certain infections or injuries. As a result, we observed the variation of cytokine secretion among the analyzed groups and samples. IL-6 was unexpectedly detected in higher concentrations in the spleen and thymus of SAM-R1 mice than in senescent mice, and RAPA treatment further increased the level of this cytokine in both lymphoid organs. A similar phenomenon was found at the genetic level by Rokytová et al. (2020), who suggested the inhibitory capacity of RAPA on the Th2 profile of lymphocytes, due to the role of mTOR in modulating this profile (54). On the other hand, the amount of TNF-α in the serum and spleen samples of the SAM-P8 groups was higher, although not significantly different. Our findings suggest that the accelerated aging process in the SAM-P8 model is not related to the elevated secretion of SASP, a finding that reveals the complexity of the aging process, which does not follow a defined and linear time-dependent pattern, but rather varies between tissues and organs and cannot be determined by just one isolated marker, given that the SAM-P8 model is very well characterized and studied in conditions of cerebral and vascular aging (55–60).

In addition to the inflammatory cytokines above, IL-7 and IL-15 levels were also determined in bone marrow. In this microenvironment, senescence affects multiple cells within the hematopoietic lineage, resulting in a gradual loss of self-renewal and differentiation capacity, both of which are important in immune function (61). Ikuta et al. (2022) also detected these interleukins in samples of bone marrow, lymph nodes, and thymus, and found different expressions in each tissue analyzed (62). As a result, we observed that SAM-P8 mice treated with RAPA presented increased levels of these cytokines compared to their respective control groups. These interleukins are the main cytokines produced by medullary stromal cells and play important roles in the immune system, with IL-7 mainly involved in the development and survival of T and B cells. On the other hand, IL-15 plays a fundamental role in the activation and proliferation of T and B cells (62, 63), which means that the increase in its expression caused by RAPA treatment may be involved in the rejuvenation of hematopoietic stem cells from C57BL/6 mice described by Chen et al. (2009), where they show the ability of RAPA to restore lymphocyte populations and response to vaccines by B (64).

The thymus is the main organ for the maturation and development of immunocompetent T lymphocytes (65). This organ also undergoes changes throughout the aging and immunosenescence process, with a considerable reduction in the size of thymic structures, such as a decrease in the medullary zone, an increase in the cortical zone, and perivascular spaces. Despite these changes, the thymus still functions, however, with reduced capacity and effectiveness (61). In our histological analysis, we observed faster thymic involution in SAM-P8 mice. In addition to this data, the population of thymic cells in these mice was much lower compared to SAM-R1 animals – results that reinforce the senescent phenotype of this model (42) and confirm that the dose of RAPA used was low enough not to affect the structure of this and other lymphoid organs evaluated, but high enough to influence the subpopulations of lymphocytes in the spleen. On the other hand, we did not observe histological changes or differences in the cellularity of the bone marrow between the groups. In the evaluation of the myelogram, the percentage of myeloid progenitor cells was higher than lymphoid cells, as expected. Harrison et al. (1978) showed that senescence in bone marrow cells of old mice promotes the depletion of the renewal capacity in hematopoietic stem cells (HSCs) (66). As described in various studies, during the aging process, the number of myeloid cells exceeds that of lymphoid cells, both in mice and humans (67, 68). This HSCs relationship between young and old organisms has been characterized by several other changes, including genetic modifications and increased production of reactive oxygen species (69).

In the histological investigation of the spleen, we did not find any morphological differences between the groups of mice from both lineages. However, we found some discrepancy in the total number of splenocytes, where SAM-R1 mice stand out, due to the larger size of this organ. The lymphoproliferation of spleen cells, stimulated by a polyclonal activator of T cells (38, 39), showed no changes between the groups, indicating that treatment with RAPA did not compromise the effectiveness of lymphocyte activation in any of the strains evaluated.

The immunophenotyping of spleen lymphocytes revealed a higher percentage of B lymphocytes compared to T lymphocytes. In adult mice, B lymphocytes originate in the bone marrow, where they undergo maturation and migrate to the spleen, composing approximately 40% of the cells in this organ (70, 71). Immunosenescence causes an imbalance of T cell subpopulations, promoting an increase in CD8^+^ T cells and decrease in CD4^+^ T cells and Treg cells (23, 72, 73). Neff et al. (2013) demonstrated that the dose of RAPA administered in their work (14 mg/kg) did not contribute to an increase in CD4^+^ T cells in C57BL/6 mice up to 22 months of age, unlike our results in RAPA-treated SAM-P8 mice, that may reflect an improvement in the immunological response (23). In addition, in the same work, they observed a reduction in the population of CD8^+^ T cells and Treg cells in 16-month-old mice (23). We observed similar results in SAM-P8 mice treated with RAPA, which may represent an improvement in the population inversion phenomenon of lymphocytes caused by immunosenescence, something completely unprecedented until now, even if on a modest scale – if we consider the findings obtained by Arriola et al. (2015), who proposed the intermittent protocol every 5 days that we used in this study as a way of reducing the immunosuppressive side effects of RAPA on the immune system. Despite there actually being an improvement when compared to the administration of a daily dose, its values of all lymphocyte populations evaluated in the spleen of C57BL6 mice are still lower than their untreated control group, something that we attribute to a dose of 2 mg/kg (31), which signals the use of low doses of RAPA as a better approach with better results.

Taken together, our findings help to understand the physiological characteristics of SAM-P8 and SAM-R1 mice. In the SAM-P8 model, although we observed thymic involution, one of the clear characteristics of senescence, other markers were not so clear, possibly due to the early analysis that we defined to evaluate such properties, which refer to when the animals were 12 and 20 weeks old. Despite our results collaborating with future research with these animals, evaluating other ages becomes necessary.

Our discoveries about the effects of RAPA on the process of immunosenescence were also presented, with interesting data related to the populations of lymphocytes important to the adaptive immune response and the secretion of cytokines that directly collaborate with the maturation and proliferation of these cells. Thus, future studies are necessary to elucidate the possible interactions of the beneficial effects of low doses of RAPA in animal models, as well as the changes that occur in the complex pathways of the mTOR protein and autophagy, and how both act against the signals of immunosenescence, senility, and aging, to then be able to evaluate the feasibility of using RAPA as an anti-aging treatment in humans.

## 5 Conclusions

In summary, we showed that the administration of RAPA at low doses and under a specific interval is capable of not only avoiding the immunosuppressive effects of the drug, but also reducing the populations of CD8^+^ T lymphocytes and increasing CD4^+^ T lymphocytes in the spleen in SAM-P8 mice. Since a ratio inversion of these lymphocyte populations is a mark of immunosenescence in these animals, RAPA treatment was able to alleviate this phenomenon. Furthermore, the treatment increased the production of IL-7 in the bone marrow, which is a hallmark cytokine for the development and survival of T and B lymphocytes. On the other hand, SAM-R1 animals showed opposite results, with an increase in CD8^+^ cells and a reduction in CD4^+^ T cells, in addition to an increase in IL- in the thymus and spleen, suggesting a dual action of RAPA depending on the phenotypic aging state of the animal. Finally, we cannot fail to mention the immunological changes between the lineages as an indicator that reinforces the SAM-P8 lineage as a good model for immunosenescence studies.

## Data availability statement

The raw data supporting the conclusions of this article will be made available by the authors, without undue reservation.

## Ethics statement

This study was evaluated and approved by the Ethics Committee on Animal Use (CEUA) at the School of Pharmaceutical Sciences (protocol number: CEUA/FCF/632) and at the Institute of Biomedical Sciences (protocol number: CEUA/ICB/4116171120) at University of São Paulo, Brazil. The study was conducted in accordance with the local legislation and institutional requirements.

## Author contributions

RB: Investigation, Writing – original draft. LD: Investigation, Writing – review & editing. KC: Funding acquisition, Investigation, Writing – review & editing. SB: Investigation, Writing – review & editing. ES: Investigation, Writing – review & editing. JA: Investigation, Writing – review & editing. AS-N: Conceptualization, Formal analysis, Funding acquisition, Writing – original draft. SR: Conceptualization, Formal analysis, Funding acquisition, Writing – original draft. EA: Conceptualization, Formal analysis, Writing – review & editing. JM: Conceptualization, Formal analysis, Funding acquisition, Project administration, Supervision, Writing – original draft.

## Supplementary material

Supplementary Figure 1 Gating strategy for flow cytometry analysis. The analyzes were carried out following the flowchart of lymphocytes defined by size and granularity, followed by single cells, for live and dead marking, and CD3^+^ T and CD19^+^ B lymphocytes. Within CD3^+^ lymphocytes, the analysis of subpopulations of FoxP3^+^ (Treg) lymphocytes and CD4^+^ (T helper) and CD8^+^ (T cytotoxic) lymphocytes.

Supplementary Figure 2 Leukogram of SAM-R1 and SAM-P8 mice. **(A)** total white blood cells (WBC); **(B)** total lymphocytes (Lymph); **(C)** percentage of lymphocytes (Lymph%); **(D)** total monocytes (Mon); **(E)** percentage of monocytes (Mon%); **(F)** total granulocytes (Gran); **(G)** percentage of granulocytes (Gran%). Hemogram analyses were performed after the euthanasia of the mice (animals at 20 weeks old). *n* = 8 (SAM-R1 CONTROL/RAPA groups); *n* = 7 (SAM-P8 CONTROL/RAPA groups). ***p* < 0.01 and *****p* < 0.0001.

Supplementary Figure 3 Evaluation of erythrogram in SAM-R1 and SAM-P8 mouse strains. **(A)** red blood cells (RBC); **(B)** percentage of hemoglobin (HGB); **(C)** hematocrit (HCT); **(D)** mean corpuscular volume (MCV); **(E)** red cell distribution width (RDW); **(F)** mean corpuscular hemoglobin concentration (MCHC); **(G)** mean corpuscular hemoglobin (MCH). Hemogram analyses were performed after the euthanasia of the mice (animals at 20 weeks old). *n* = 8 (SAM-R1 CONTROL/RAPA groups); *n* = 7 (SAM-P8 CONTROL/RAPA groups). ***p* < 0.01, ****p* < 0.001, and *****p* < 0.0001.
