# Supplementary figures and images for: Effects of low-dose rapamycin on lymphoid organs of mice prone and resistant to accelerated senescence
Source: Front Immunol. 2024 Mar 7;15:1310505. doi: 10.3389/fimmu.2024.1310505 (PMC10954823; doi:10.3389/fimmu.2024.1310505)

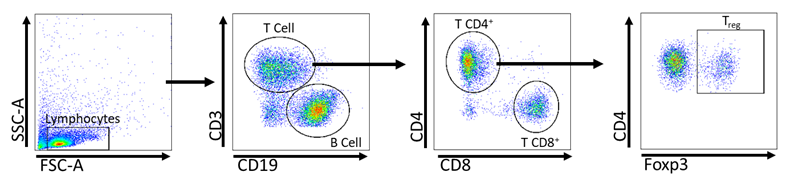

Supplement: Supplementary Figure 1 — Gating strategy for flow cytometry analysis. The analyzes were carried out following the flowchart of lymphocytes defined by size and granularity, followed by single cells, for live and dead marking, and CD3+ T and CD19+ B lymphocytes. Within CD3+ lymphocytes, the analysis of subpopulations of FoxP3+ (Treg) lymphocytes and CD4+ (T helper) and CD8+ (T cytotoxic) lymphocytes. [file Image_1.tif]

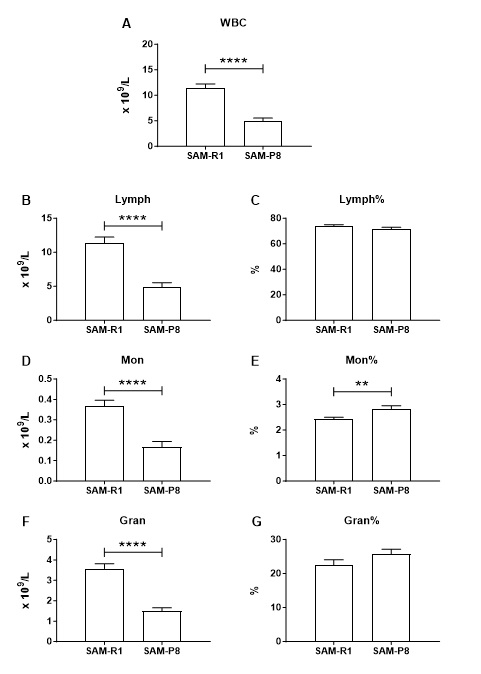

Supplement: Supplementary Figure 2 — Leukogram of SAM-R1 and SAM-P8 mice. (A) total white blood cells (WBC); (B) total lymphocytes (Lymph); (C) percentage of lymphocytes (Lymph%); (D) total monocytes (Mon); (E) percentage of monocytes (Mon%); (F) total granulocytes (Gran); (G) percentage of granulocytes (Gran%). Hemogram analyses were performed after the euthanasia of the mice (animals at 20 weeks old). n = 8 (SAM-R1 CONTROL/RAPA groups); n = 7 (SAM-P8 CONTROL/RAPA groups). **p < 0.01 and ****p < 0.0001. [file Image_2.tif]

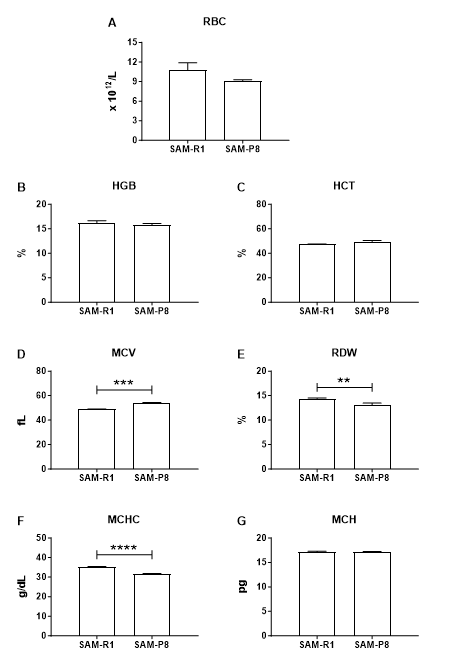

Supplement: Supplementary Figure 3 — Evaluation of erythrogram in SAM-R1 and SAM-P8 mouse strains. (A) red blood cells (RBC); (B) percentage of hemoglobin (HGB); (C) hematocrit (HCT); (D) mean corpuscular volume (MCV); (E) red cell distribution width (RDW); (F) mean corpuscular hemoglobin concentration (MCHC); (G) mean corpuscular hemoglobin (MCH). Hemogram analyses were performed after the euthanasia of the mice (animals at 20 weeks old). n = 8 (SAM-R1 CONTROL/RAPA groups); n = 7 (SAM-P8 CONTROL/RAPA groups). **p < 0.01, ***p < 0.001, and ****p < 0.0001. [file Image_3.tif]

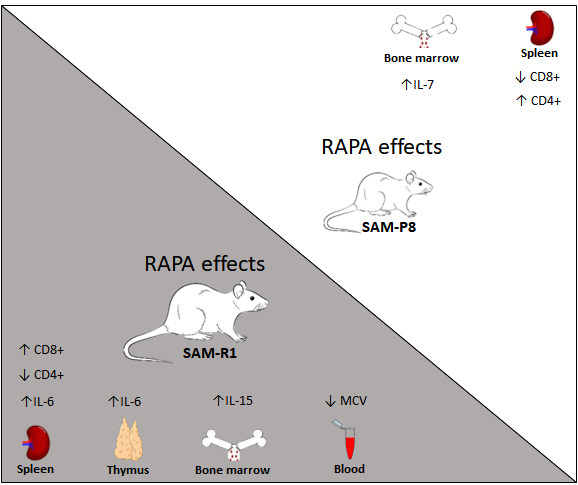

Supplement: Supplementary file 4 [file Image_4.tif]
