# Supplementary material for: Effects of low-dose rapamycin on lymphoid organs of mice prone and resistant to accelerated senescence
Source: Front Immunol. 2024 Mar 7;15:1310505. doi: 10.3389/fimmu.2024.1310505 (PMC10954823; doi:10.3389/fimmu.2024.1310505)
Supplement: Supplementary file 5 [file Table_1.docx]

**Supplementary Table 1 – Hematological parameters.**

| **Parameter** | **Reference value** |
| --- | --- |
| WBC | 0.8-0.6 x 10^12^/L |
| Lymph | 0.7-5.7 x 10^9^/L |
| Mon | 0.0-0.3 x 10^9^/L |
| Gran | 0.1- 1.8 x 10^9^/L |
| Lymph% | 55.8-90.6% |
| Mon% | 1.8- 6.0% |
| Gran% | 8.6- 38.9% |
| RBC | 6.36-9.42 x 10^12^/L |
| HGB | 11.0-14.3 g/dL |
| HCT | 34.6-44.6% |
| MCV | 48.2-58.3 fL |
| MCH | 15.8-19.0 pg |
| MCHC | 30.2-35.3 g/dL |
| RDW | 13.0-17.0% |

**Supplementary Table 1:** Hematological parameters: red blood cells (RBC), hemoglobin (HGB), hematocrit (HCT), mean corpuscular volume (MCV), mean corpuscular hemoglobin (MCH), mean corpuscular hemoglobin concentration (MCHC), red cell distribution width (RDW), leukocytes (WBC), lymphocytes (Lymph), monocytes (Mon), granulocytes (Gran), and the percentage of lymphocytes (Lymph%), monocytes (Mon%), and granulocytes (Gran%).
